# Injectable hydrogels doped with PDA nanoparticles for photothermal bacterial inhibition and rapid wound healing *in vitro*†

**DOI:** 10.1039/d3ra08219a

**Published:** 2024-01-17

**Authors:** Ying Wei, Junhua Fu, Enrui Liu, Junru Gao, Yaqing Lv, Zhenlu Li

**Affiliations:** a Department of Operating Room, The Affiliated Hospital of Qingdao University 266003 Qingdao China weiy8812@163.com Fuyanwangjiayun@163.com; b Department of Emergency Surgery, The Affiliated Hospital of Qingdao University 266003 Qingdao China lizhenlu1231@163.com liuenrui1992@163.com; c Department of Outpatient, The Affiliated Hospital of Qingdao University 266003 Qingdao China qdbzr@126.com uuto2004@126.com

## Abstract

The difficulty of wound healing due to skin defects has been a great challenge due to the complex inflammatory microenvironment. Delayed wound healing severely affects the quality of life of patients and represents a significant economic burden for public health systems worldwide. Therefore, there is an urgent need for the development of novel wound dressings that can efficiently resist drug-resistant bacteria and have superior wound repair capabilities in clinical applications. In this study, we designed an adhesive antimicrobial hydrogel dressing (GMH) based on methacrylic-anhydride-modified gelatin and oxidized hyaluronic acid formed by Schiff base and UV-induced double cross-linking for infected wound repair. By inserting PDA nanoparticles into the hydrogel (GMH/PDA), the hydrogel has the capability of photothermal conversion and exhibits good photothermal antimicrobial properties under near-infrared (NIR) light irradiation, which helps to reduce the inflammatory response and avoid bacterial infections during the wound healing process. In addition, GMH/PDA hydrogel exhibits excellent injectability, allowing the hydrogel dressings to be adapted to complex wound surfaces, making them promising candidates for wound therapy. In conclusion, the multifunctional injectable GMH/PDA hydrogel possesses high antimicrobial efficiency, antioxidant properties and good biocompatibility, making them promising candidates for the treatment of infected skin wounds.

## Abstract 2

Schematic illustration of the network structure of and wound repair by GMH/PDA hydrogel.

## Graphical abstract

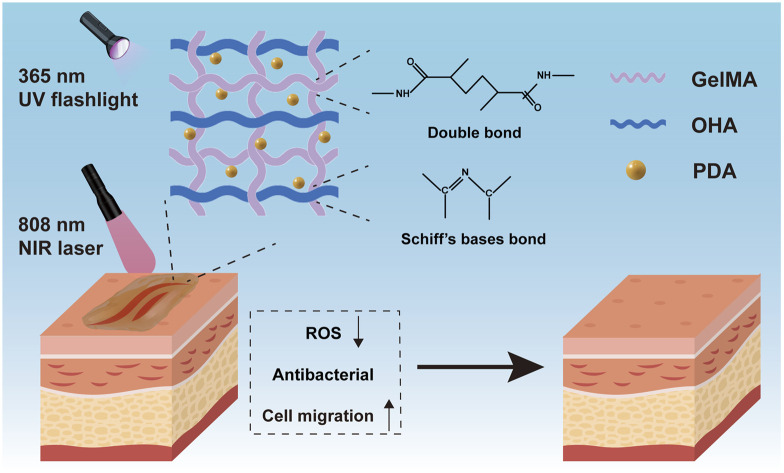

## 1 Introduction

The skin is the primary barrier against microbial invasion and is susceptible to damage from external exposure.^1^ The healing process of damaged skin is highly complex.^2^ It should be noted that bacterial infection at the wound site complicates the repair process.^3^ Presently, commercially available creams and ointments for treating infected wounds have limitations, including limited antimicrobial effectiveness, low solubility, and ineffectiveness against biofilms.^4,5^ Thus, there is an urgent need to develop a treatment that can safely and effectively enhance the healing of infected wounds.

Various strategies—including electrostatically spun fibers, metal–organic frameworks and hydrogels—have been developed to promote wound healing, among which hydrogels have attracted a lot of attention.^6–8^ Hydrogel, a polymer with a three-dimensional network structure, has emerged as a highly promising biomaterial for wound healing owing to its exceptional biocompatibility, degradability, and ability to absorb wound exudate.^9^ Nevertheless, conventional hydrogels lack antimicrobial properties, rendering the wound site vulnerable to microbial attack.^10,11^ In addition, most hydrogels have poor injectability, making it difficult to accommodate wound irregularities.^12^ Therefore, the ideal wound healing hydrogel dressing should have a combination of injectability, antimicrobial properties and promotion of cell migration.

Gelatin, a protein extracted from animal connective tissue, has good biocompatibility and biodegradability, making it an ideal material for the preparation of biomedical materials.^13,14^ Firstly, gelatin possesses excellent bio-adhesion properties that promote cell attachment and proliferation, contributing to tissue repair and regeneration.^15,16^ Secondly, gelatin exhibits excellent biocompatibility with human tissues, avoiding any immune reactions or rejection.^14^ Additionally, gelatin exhibits good biodegradability, eliminating the need for secondary surgical procedures.^17^ Therefore, gelatin has a wide range of applications in the fields of tissue engineering, drug delivery systems and trauma repair.^18–20^ Hyaluronic acid (HA), a natural polysaccharide widely found in the connective tissues, cartilage, and skin of the human body, exhibits good biocompatibility and biodegradability.^21–23^ Notably, the molecular structure of HA contains numerous hydroxyl and carboxyl functional groups that enable it to exhibit excellent hydration ability, and form a protective hydrogel layer that promotes wound healing and regeneration.^24,25^ Additionally, HA promotes cell proliferation and migration, thereby accelerating wound healing processes.^26,27^ However, the application of hyaluronic acid faces some challenges, such as biostability and adhesion issues, and further modifications are needed to facilitate its application and development in biomedical materials.^28,29^

Polydopamine (PDA) is a biopolymer compound widely found in nature.^30^ It is a polymer formed by the oxidative polymerization reaction of dopamine molecules with a large number of tyrosine units in its structure.^31^ PDA has excellent antioxidant properties and biocompatibility, and can be used as a carrier for antioxidants and drugs to promote their stability and biological activity.^32–34^ In recent years, researchers have discovered that PDA has an excellent photothermal property, *i.e.*, the ability to generate heat in the presence of light.^35,36^ This special property makes PDA an ideal material for photothermal conversion. These properties give PDA a wide range of application prospects in the fields of photothermal therapy, photothermal conversion and photothermal catalysis.^37–39^ Therefore, PDA has broad application prospects in the fields of biomedicine, material science and nanotechnology.

**Scheme 1:**
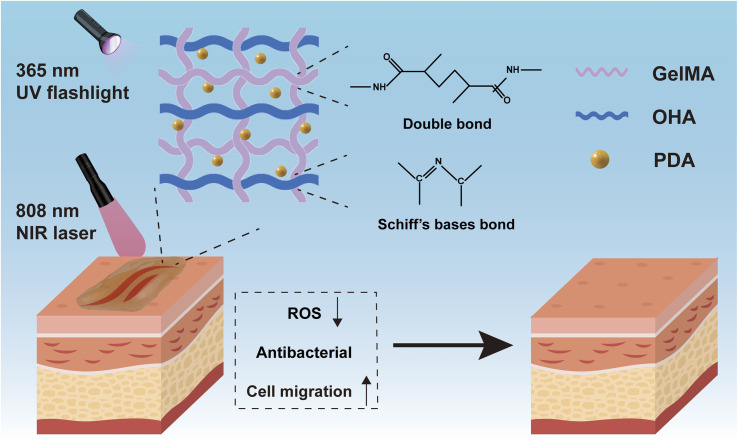
Schematic illustration of the network structure of and wound repair by GMH/PDA hydrogel.

In this study, we have engineered an injectable hydrogel, denoted GMH/PDA, comprising GelMA/oxidised HA (OHA)/PDA, which exhibits photothermal antimicrobial properties to enhance wound healing acceleration. The GMH/PDA hydrogel is a dual-network structure, formed by cross-linking methacrylic-acid-modified gelatin and oxidized hyaluronic acid through Schiff base bonds, followed by additional polymerization under ultraviolet (UV) irradiation. The incorporation of PDA nanoparticles into the hydrogel confers upon it the ability to harness photothermal therapy. Dual-network injectable hydrogels offer an enhanced degree of cross-linking, elevating their mechanical properties. Additionally, unlike conventional single-component photoinitiated hydrogels, they assist in minimizing hydrogel loss from the wound before UV light curing (Scheme 1). Moreover, the antioxidant and photothermal antimicrobial properties of the hydrogel serve to mitigate inflammatory responses and prevent infections throughout the wound healing process. Experimental results from cell migration assays confirm that the GMH/PDA hydrogel substantially enhances cell migration rates, thereby expediting the wound healing process. Consequently, the multifunctional injectable GMH/PDA hydrogel exhibits remarkable potential for advancing wound healing and holds broad clinical applicability.

## 2 Experimental section

### 2.1. Materials

Sodium hyaluronate (average molecular weight 1–1.5 million) was purchased from Shanghai Yuanye Bio-Technology Co., Ltd. Sodium hydroxide (96%) and methacrylic anhydride were obtained from Aladdin Reagent Co., Ltd. (Shanghai, China). Sodium periodate (98%) and type A gelatine were obtained from Sinopharm Chemical Reagent Co., Ltd. (Shanghai, China). Ethylene glycol was ordered from General-Reagent Co., Ltd. (Shanghai, China). Dopamine hydrochloride (dopamine, 98%) and polyvinylpyrrolidone (MW = 360k) were purchased from J&K Technology Co., Ltd. Irgacure 2959 photoinitiator was purchased from Aladdin Reagent Co., Ltd. (Shanghai, China). Dulbecco's Modified Eagle Medium was acquired from Corning Co., Ltd. (Shanghai, China). Mice fibroblast cells and human immortalized epidermal (HaCat) cells were bought from the Institute of Biochemistry and Cell Biology (Shanghai, China). A live/dead cell dichromatic stain kit was ordered from Maokang Biotechnology Co., Ltd. (Shanghai, China). Cell Counting Kit-8 (CCK-8) was obtained from Beyotime Biotechnology (Shanghai, China). All reagents were used without further purification.

### 2.2. Preparation of methacrylic-anhydride-grafted gelatin

A 10% w/v solution of type A gelatin was dissolved in deionized water with magnetic stirring at 50 °C. Methacrylic anhydride was added gradually drop by drop to the gelatin solution using a microsyringe pump at an injection rate of 35 mL h^−1^. The microsyringe pump was stopped when the final MA-to-gel ratio in the mixed solution reached 0.1 mL to 1 g. The mixture was stirred at 50 °C for an additional 4 h to ensure complete reaction. The reacted solution was purified by dialysis in deionized water using a 14 kDa cut-off dialysis bag (Viskase, USA) over 4 d to eliminate any unreacted methacrylic anhydride. The product was lyophilized to yield GelMA.

### 2.3. Preparation of OHA

Initially, 2 g of HA were fully dissolved in 200 mL of deionized water at 50 °C. Subsequently, 1.5 g of NaIO_4_ powder were added, and the reaction was conducted at 50 °C for 12 h under protection from light. Termination of the reaction was achieved by adding 2 mL of ethylene glycol. The reaction mixture was dialyzed in deionized water for 2 d utilizing a dialysis membrane with a molecular weight cutoff of 3500 Da (Viskase, USA) and subsequently lyophilized with a lyophilizer (Biocool FD-1A-50, China). The final product (OHA powder) was stored at room temperature for further use.

### 2.4. Preparation of PDA nanoparticles

First, 90 mg of dopamine (DA) were dissolved in 40 mL of deionized water. Once complete dissolution had occurred, 0.38 mL (1 M) of NaOH solution was introduced, and the mixture was then transferred to an ice-water bath and stirred for 24 h. Then 1 g of PVP was added to the mixed solution, and stirring was maintained for 4 h. After the reaction was complete, the mixture was subjected to centrifugation and washed three times with deionized water at 15 000 rpm for 10 min each time.

### 2.5. Preparation of the hydrogels

To prepare the GelMA/OHA/PDA + UV hydrogel (GMH/PDA), OHA (50 mg mL^−1^) and GelMA (200 mg mL^−1^) were dissolved into a homogeneous solution. Then, PDA solution (0.5 mg mL^−1^) was added to the above mixture. Subsequently, Irgacure 2959 (10 mg mL^−1^) was added, and ultraviolet (UV) irradiation (365 nm, 260 mW cm^−2^) was employed for 60 s. Control hydrogel samples were prepared similarly: (1) GelMA, (2) GelMA/OHA (GMA) and (3) GelMA/OHA + UV (GMH).

### 2.6. Characterization of GelMA, OHA and hydrogels

The internal microstructures of the GMA, GMH and GMH/PDA hydrogels were observed by field emission scanning electron microscopy, FESEM (Zeiss Sigma 300, Germany). A digital camera was used to record the formation of the hydrogels. Using Irgacure 2959 as a photoinitiator, when irradiated with UV light, GelMA can be rapidly crosslinked with OHA and PDA to form GMH/PDA hydrogel. To verify the successful oxidation of HA to OHA by sodium periodate, nuclear magnetic resonance spectroscopy (NMR, Bruker 400 MHz, Germany) was used to detect the hydrogen atoms of the aldehyde group (–CHO).

### 2.7. Adhesion property of the hydrogels

Hydrogel adhesion was evaluated through shear experiments. Subsequently, pig skin underwent fat removal and PBS washing; then, it was cut with scissors into 10 mm × 20 mm rectangular sheets. During testing, two pig skin pieces were partially overlapped, and the pre-prepared hydrogel was applied between them. Slow stretching occurred on a universal testing machine at a speed of 10 mm min^−1^ under a 0.1 N external force until separation of the pig skin. The stress–strain curve was documented throughout the test. Additionally, the adherence of the GMH/PDA hydrogel to the specimen surface was observed.

### 2.8. Mechanical property evaluation

Dynamic rheological studies of GMA, GMH and GMH/PDA hydrogels were carried out using a rotational rheometer (MARS III HAAKE). Time-scan oscillation tests were performed at 1 Hz frequency, 1 mm gap and 10% strain. Specifically, parallel plates (P20 TiL, 20 mm diameter) were injected with the corresponding hydrogel precursor solutions, and the gap was adjusted to 1 mm for strain scanning, to obtain the linear response of the above solutions at 37 °C. The frequency scan measurements of the hydrogels were expressed using the final storage modulus (*G*′) and the final loss modulus (*G*′′). The gel point of the hydrogel can be determined when *G*′ exceeds *G*′′. The mechanical properties of the hydrogels were evaluated using a universal material tester (Zwick Roell Z2.5) with a sensor of 2.5 kN. For the compression tests, GMA, GMH and GMH/PDA hydrogels were separately molded in a mold (10 mm in diameter and 3 mm in height). The compression modulus was recorded in a linear fit to the stress–strain curve (rate: 1 mm min^−1^ and strain range: 20–40%).

### 2.9. Swelling kinetics of the hydrogels

The swelling kinetics of the GMA, GMH and GMH/PDA hydrogels were tested in simulated body fluid (SBF) and deionized water. The freeze-dried GMA, GMH and GMH/PDA hydrogels were weighed as *W*_0_. Then, the GMA, GMH and GMH/PDA hydrogels were immersed in 10 mL of SBF and deionized water (37 °C), respectively. The weights *W*_*t*_ of the different hydrogels were recorded at different time points (5, 10, 20, 40, 60, 80, 100 and 120 s). The dissolution rates of GMA, GMH and GMH/PDA hydrogels were calculated from eqn (1).1
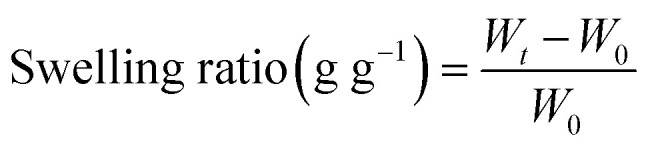


### 2.10. Porosity of the hydrogels

The hydrogel porosity was determined as follows: the freeze-dried hydrogel was weighed and soaked in 5 mL of ethanol for 30 min. Then the hydrogel was removed from the ethanol, the surface water was wiped dry, and the hydrogel was weighed again. The porosity was calculated according to eqn (2):2
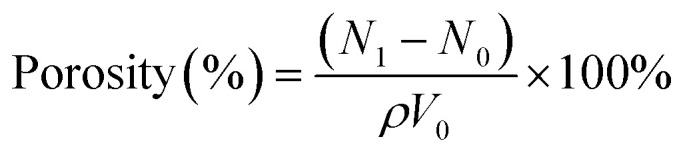


In the equation, *N*_0_ denotes the mass of the hydrogel before it is immersed in ethanol and *N*_1_ denotes the mass of the hydrogel after it is immersed in ethanol. *ρ* is the density of the ethanol (0.785 g cm^−3^) and *V*_0_ is the volume of the hydrogel.

### 2.11. *In vitro* degradation

The lyophilized GMA, GMH and GMH/PDA hydrogels (*W*_d_) were weighed and separately added to 10 mL of SBF. The samples were continuously shaken in a constant-temperature air-bath shaker at 37 °C for 56 days. The mock body fluid was changed every other day. Finally, the hydrogels were removed from the incubation medium, gently rinsed with SBF, lyophilized and weighed again (*W*_*t*_) to calculate the mass residual rate according to eqn (3):3
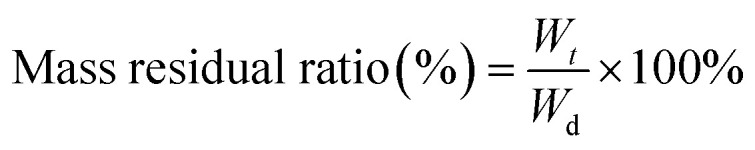


### 2.12. *In vitro* antibacterial activity

The bacteriostatic properties of the GMH/PDA hydrogel were verified by liquid antimicrobial assay. *Escherichia coli* (*E. coli*) and *Staphylococcus aureus* (*S. aureus*) were separately added to Luria–Bertani (LB, Shanghai Yuanye Biotechnology Co., Ltd.) liquid medium. The experimental groups were divided into GelMA, GMH, GMH/PDA and GMH/PDA + NIR groups. For the GelMA, GMH and GMH/PDA groups, the prepared cylindrical hydrogels (11 mm in diameter and 3 mm in thickness) were placed in LB medium and incubated with bacteria for 12 h (37 °C). For the GMH/PDA + NIR group, the hydrogel-containing bacterial solution was placed in a 55 °C incubator for 5 min and then transferred to a 37 °C incubator to continue incubation for 12 h. The bacterial suspensions (*A*_s_) of each experimental group were determined using a UV-vis-NIR spectrophotometer (Lambda 25, PerkinElmer, USA). Absorption at 625 nm (*A*_c_) of pure bacterial suspensions was used as a positive control. The inhibition ratio was calculated according to the eqn (4). In the solid antimicrobial test, the bacterial fluids of different treatments in the above groups were coated on LB medium and incubated at 37 °C for 12 h, and then photographed with a digital camera.4
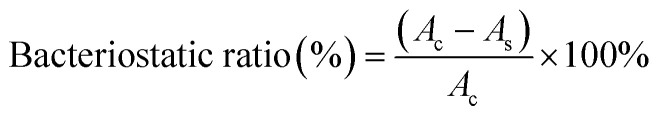


### 2.13. Photothermal capacity of the hydrogel

First, 500 mg of hydrogel was placed in a container and exposed to UV irradiation to create a GMH/PDA hydrogel. Before commencing the experiment, 200 μL of PBS was introduced into the hydrogel-containing container to mimic the physiological conditions. To examine how NIR power density influences the GMH/PDA hydrogel, GMH/PDA hydrogel was irradiated with 808 nm NIR at power densities of 0.8 W cm^−2^, 0.9 W cm^−2^, and 1.0 W cm^−2^ for 3 min at room temperature. An infrared thermography camera (FLIR E60, Wilsonville, OR, USA) was employed to monitor changes in hydrogel temperature, and readings were taken every 10 s.

The photothermal stability of the GMH/PDA hydrogel was assessed through five “ON/OFF” heating and cooling cycles. In summary, the GMH/PDA hydrogel was exposed to 808 nm NIR at 1.0 W cm^−2^, and temperature changes were monitored. The NIR irradiation was terminated after 5 min. Subsequently, the hydrogel was allowed to cool naturally at room temperature for 5 min, followed by another exposure to 808 nm NIR at 1.0 W cm^−2^. This cycle was repeated five times.

To assess the photothermal conversion efficiency of the GMH/PDA hydrogel, we initially measured their absorbance at 808 nm using UV-vis-NIR spectroscopy (N4-N4s INESA). Subsequently, 500 mg of GMH/PDA hydrogel was exposed to NIR at 808 nm (1.0 W cm^−2^) for 5 min. Following the cessation of NIR irradiation and allowing for natural cooling, temperature fluctuations were recorded at 20 s intervals. The photothermal conversion efficiency of the hydrogels was determined using the formula:5
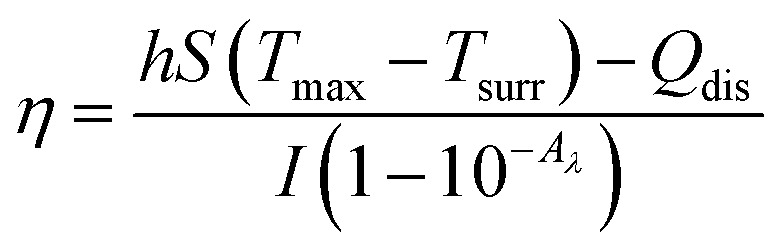
where *h* is the heat transfer coefficient, *S* is the surface area of the NIR-irradiated GMH/PDA hydrogel, *T*_max_ is the maximum temperature of the GMH/PDA hydrogel, *T*_surr_ is the room temperature at the time of the test, *I* is the NIR power, *A*_*λ*_ is the absorbance of the absorbent sample at 808 nm, and *Q*_dis_ is the heat generated by the PBS under NIR irradiation. In addition, *hS* can be obtained from the following formula:6
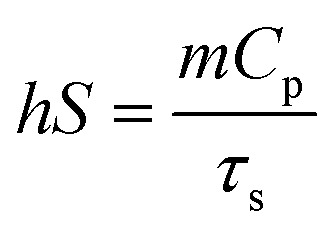
where *m* and *C*_p_ are the mass and heat capacity of H_2_O, respectively, and *τ*_s_ is the time constant determined from the fitted curve of the time of GMH/PDA hydrogel during the cooling process to −ln(*θ*).

### 2.14. Antioxidant capacity of the hydrogel

Nitrogen-centered radicals (DPPH) and oxygen-centered radicals (PTIO) were selected to assess the antioxidant capacity of the GMH/PDA hydrogel. In brief, lyophilized GMH/PDA hydrogel was immersed in 5 mL of either DPPH or PTIO working solution at 37 °C. After 3 min incubation with the DPPH solution, the hydrogel was extracted, and the absorbance of the supernatant was analyzed between 400 and 800 nm using UV-vis-NIR spectroscopy. Likewise, the hydrogel was exposed to the PTIO solution for 30 min, followed by removal and measurement of the supernatant's absorbance at 400–800 nm using UV-vis-NIR spectroscopy. Under identical conditions, a control group without the addition of hydrogel was established as a blank control. The hydrogel's antioxidant capacity was determined using the equation below:7
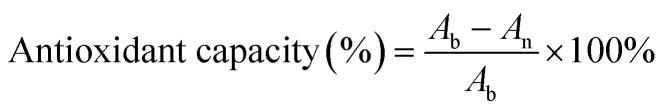
where *A*_b_ is the absorbance of the blank group and *A*_n_ is the absorbance of the supernatant at the characteristic absorption. The characteristic absorption of the DPPH group was at 517 nm and that of the PTIO was at 557 nm in UV-vis-NIR spectroscopy.

### 2.15. *In vitro* cytotoxicity testing

L929 cells were selected to test cell safety. Briefly, L929 cells were grown in DMEM medium containing 10% FBS and 1% antipenicillin and streptomycin. The cells were inoculated in 96-well plates at a density of 8000 cells per well when the cells were in the logarithmic growth phase. The prepared hydrogel samples were immersed in DMEM to obtain an extract of the hydrogel. The concentrations of the extracts were 0, 2.5, 5, 10, and 20 mg mL^−1^. Cells were incubated overnight at a constant temperature of 37 °C and 5% CO_2_ in a thermostat incubator. Next, the original medium was removed and replaced with the corresponding hydrogel extract to continue the incubation for 24 h and 48 h. At predetermined time points, CCK-8 working solution was added to each well according to the manufacturer's instructions and incubation was continued at 37 °C for 2 h. The absorbance of each well at 450 nm was recorded using a multifunctional enzyme labeler. The cell viability was calculated according to the formula in the literature.

Cell live–dead staining kits were used to assess the cytocompatibility of the hydrogels. The culture conditions and experimental grouping were similar to those for the CCK-8 assay. The working solution was added to each well according to the manufacturer's instructions and incubation was continued for 15 min. Cell morphology and coloration were then observed under a fluorescence microscope.

### 2.16. Hemolytic test

Blood samples were collected from anesthetized rats using cardiac blood sampling and placed in anticoagulation tubes. The collected fresh blood (1 mL) was centrifuged (3000 rpm, 5 min) five times to obtain red blood cells. The obtained erythrocytes were resuspended with PBS and diluted to a concentration of 5% (w/v). The diluted erythrocyte suspension was mixed with different samples (the final concentrations of the GMH/PDA hydrogel were 2.5, 5, 10, and 20 mg mL^−1^) and incubated for 3 h. Positive controls were H_2_O and negative controls were PBS. Afterwards, the mixed solution was centrifuged (3000 rpm, 10 min) to collect the supernatant. A digital camera was used to record a photograph of the supernatant and determine its absorbance at 540 nm. The ratio of hemolysis was calculated for each group according to the formula:8
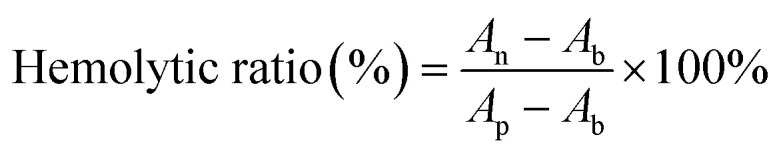
*A*_n_ is the absorbance of the experimental group. *A*_b_ is the absorbance of the negative control group. *A*_p_ is the absorbance of the positive control group.

### 2.17. Cell migration test

The ability of the hydrogels to promote cell migration was determined using a cell scratch assay. L929 cells in logarithmic growth phase were inoculated at a density of 1 × 10^6^ in a 6-well plate overnight to form a monolayer of cells growing all over the bottom of the plate. Guided by a straight edge, a 10 μL tip was used to slice through a monolayer of cells to create a simulated wound. Cells were washed three times with PBS to remove cellular debris, after which an extract of hydrogel (GelMA, GelMA/PDA, GMH and GMH/PDA) at a concentration of 10 mg mL^−1^ was added to each well, and medium without hydrogel extract was used as a control. At predetermined times, the ratio of cell migration was recorded using an inverted phase contrast microscope, and the migration ratio was calculated from the following equation:9
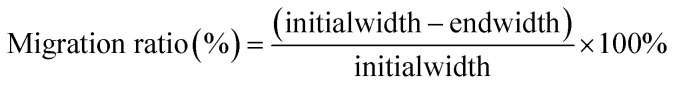


### 2.18. Statistical analysis

Differences were evaluated using the one-way analysis of variance (ANOVA), *p < 0.05, **p < 0.01, ***p < 0.001, ns: not specified. The sample size was three (*n* = 3).

## 3 Results and discussion

### 3.1. Characterization of GelMA, OHA and hydrogels

**Fig. 1:**
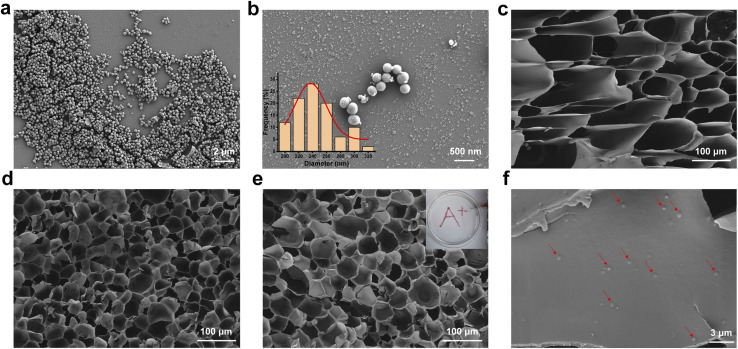
FESEM images of (a) and (b) PDA (inset: histogram distribution of the particle size of PDA), (c) GMA, (d) GMH, and (e) GMH/PDA; (f) PDA in GMH/PDA hydrogel (red marks).

The microstructures of the PDA, GMA, GMH and GMH/PDA hydrogels were observed by FESEM. The FESEM photographs showed that the different hydrogels had a porous internal structure. The FESEM images show that the PDA nanoparticles exhibit a uniform spherical shape (Fig. 1a and b). The synthetic PDA diameter was calculated to be 255.4 ± 31.3 nm (inset to Fig. 1b). The amino group in GelMA can form a Schiff base group with the aldehyde group in OHA, turning the solution into a hydrogel (GMA, Fig. 1c). In addition, the polymerization reaction of GelMA can be initiated by irradiation with UV light in the presence of the photoinitiator Irgacure 2959, forming a dual-network hydrogel (GMH, Fig. 1d). Addition of PDA had less effect on the structure of GMH hydrogels (Fig. 1e). Interestingly, the GMH/PDA hydrogel exhibits excellent injectable properties and can be injected to form customizable patterns (inset to Fig. 1e). More importantly, the internal pore density increased with increasing cross-linking degree. This was confirmed by the determination of hydrogel porosity. The porosity of the GMH hydrogel (46.0 ± 5.2%) was lower than that of GMA (62.7 ± 4.1%, Fig. S1†), which was attributed to the higher degree of cross-linking of GMH, which resulted in smaller pore diameters and lower porosities for GMH than for GMA hydrogel. The porosity of GMH/PDA hydrogel was similar to that of GMH hydrogel, at 47.7 ± 2.6%. These dense pore structures help to facilitate the transportation of intercellular fluid, which promotes wound healing.

**Fig. 2:**
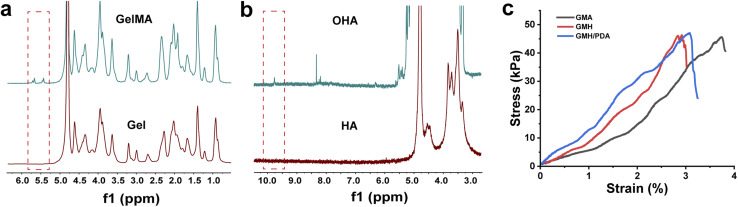
(a) ^1^H NMR spectra of GelMA and gel; (b) ^1^H NMR spectra of OHA and HA. (c) Strain–stress curves for overlapping shear tests.

The successful synthesis of GelMA was confirmed by ^1^H NMR. As shown in Fig. 2a, a signal corresponding to –CH_2_ appeared in the spectrum of GelMA in the range of 5.3–5.7 ppm. In addition, HA was also confirmed to be successfully oxidized with the appearance of a new resonance peak in the chemical shift range of 9.0–10.0 ppm, which can be assigned to the –CHO proton (Fig. 2b).

### 3.2. Swelling kinetics of hydrogels

The swelling kinetics of GMA, GMH and GMH/PDA hydrogels in different solutions were investigated (Fig. S2†), reaching 86.7 ± 7.2, 74.4 ± 4.7 and 72.3 ± 3.8 g g^−1^ in DI water, and 45.0 ± 3.1, 38.1 ± 1.6 and 32.9 ± 3.2 g g^−1^ in SBF after 20 min of storage, respectively. The swelling rates of GMH and GMH/PDA hydrogels were lower than that of the GMA hydrogel due to the higher degree of cross-linking of GMH and GMH/PDA. In addition, GMH/PDA hydrogel maintained its structural integrity and stability after sufficient swelling, which is important for wound healing in clinical practice.

### 3.3. *In vitro* biodegradation

During the wound healing process, hydrogels should be able to adhere to the wound tissue for a period of time. Therefore, the degradation ratio of hydrogels is an important factor affecting their clinical application. First, the *in vitro* degradation behavior of GMA, GMH and GMH/PDA hydrogels was investigated (Fig. S3†). It was found that GMH and GMH/PDA hydrogels degraded at similar rates after 56 days of degradation in SBF, maintaining approximately 12.3% and 12.6% of their original mass. In contrast, GMA was almost completely degraded after 7 days. The difference in degradation ratios can be attributed to differences in the degree of cross-linking.

### 3.4. Evaluation of adhesion and mechanical properties of hydrogels

To extend its duration within the wound, the hydrogel must possess adhesive properties. As illustrated in Fig. 2c, this interaction occurs between the aldehyde group of the hydrogel and the skin's amino group, giving the hydrogel its adhesive nature. GMA (45.6 ± 2.2 kPa), GMH (46.3 ± 1.9 kPa), and GMH/PDA (46.8 ± 2.4 kPa) hydrogels exhibit comparable maximum shear strengths. GMA displayed increased strain in the shear test owing to its minimal cross-linking. Adhesion experiments verified the prolonged adherence of GMH/PDA hydrogel to the wound surface (Fig. S4†).

**Fig. 3:**
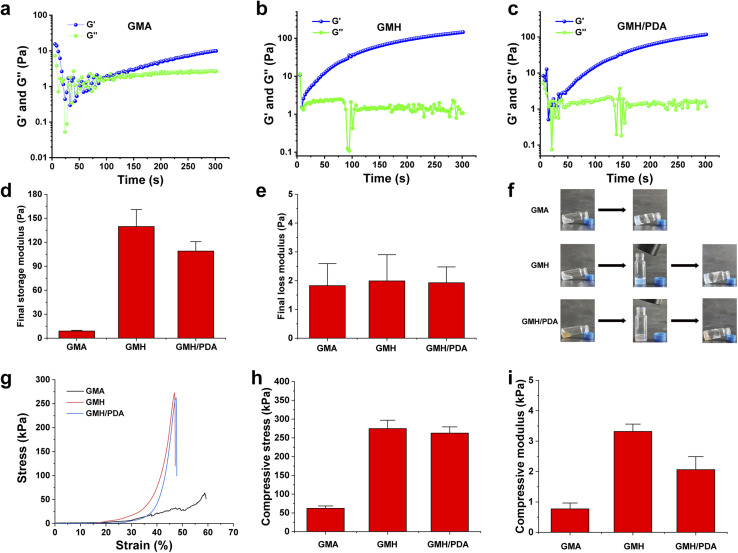
Dynamic time-scan rheological analysis of (a) GMA, (b) GMH and (c) GMH/PDA hydrogels. (d) The final *G*′ and (e) *G*′′ of different hydrogels. (f) Photographs of the formation process of GMA, GMH and GMH/PDA hydrogels. (g) Compressive strain–stress curves of different hydrogels. (h) The average compressive stress and (i) compressive modulus of different hydrogels.

The gelation process of the GMA, GMH and GMH/PDA hydrogels was investigated by rheological analysis (Fig. 3a–c). Intersection of the *G*′ and *G*′′ curves can be observed from the rheological curves of GMA, GMH and GMH/PDA hydrogels. This result indicates the transformation of the hydrogel precursor from sol to gel. Statistically, the gel times of the GMA, GMH and GMH/PDA hydrogels were 75.6 ± 0.9, 8.9 ± 0.4 and 22.2 ± 1.7 s, respectively. The *G*′ of the GMA, GMH and GMH/PDA hydrogels were 9.0 ± 0.9, 139.7 ± 21.2 and 109.0 ± 11.7 Pa, respectively. The *G*′′ of the GMA, GMH and GMH/PDA hydrogels were 1.8 ± 0.8, 2.0 ± 0.9 and 1.9 ± 0.5 Pa, respectively. The process of hydrogel formation was documented with a digital camera (Fig. 3f). Then, the compressive properties of the GMA, GMH and GMH/PDA hydrogels were compared (Fig. 3g). The results showed that the Schiff base crosslinked structure increased the compressive stress of the GMH and GMH/PDA hydrogels (the maximum compressive stress increased from 62.1 ± 6.3 kPa (GMA) to 274.7 ± 22.4 kPa (GMH) and 262.7 ± 16.7 kPa (GMH/PDA) (Fig. 3h)). This is due to the improved flexibility of the hydrogel after the introduction of the double bond.

### 3.5. The photothermal capacity of GMH/PDA hydrogel

**Fig. 4:**
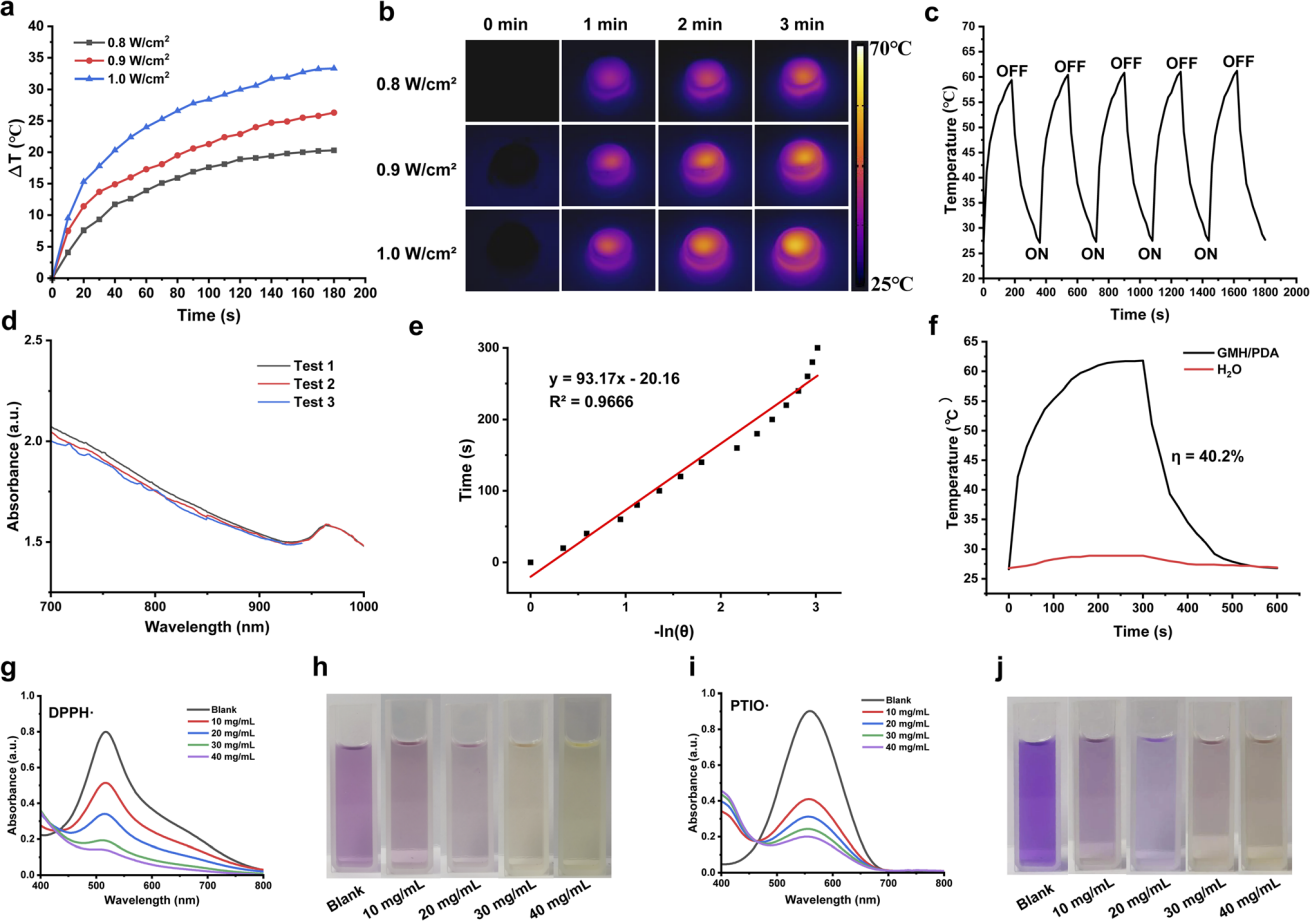
Temperature profiles (a) and infrared imaging images (b) of GMH/PDA hydrogel under 0.8, 0.9, and 1.0 W cm^−2^ NIR irradiation. (c) Temperature change of GMH/PDA hydrogel under 1.0 W cm^−2^ NIR irradiation with five “ON/OFF” cycles. (d) UV-vis-NIR absorption curves of GMH/PDA hydrogel. (e) Fitted curves for −ln(*θ*) and time. (f) Heating and cooling profiles of GMH/PDA hydrogel and deionized water under NIR laser irradiation. (g and h) UV-vis-NIR absorption spectra (g) and digital photograph (h) of DPPH removal by GMH/PDA hydrogel of different concentrations. (i and j) UV-vis-NIR absorption spectra (i) and digital photograph (j) of PTIO removal by GMH/PDA hydrogel of different concentrations.

Photothermal therapy represents a straightforward and efficacious antibacterial approach. It disrupts bacterial integrity by subjecting the bacteria to elevated temperatures, causing the melting and denaturation of cell membranes.^40^ Consequently, this results in the leakage of intracellular and extracellular material, ultimately culminating in bacterial demise. Furthermore, elevated temperatures disrupt bacterial metabolic pathways, impeding crucial enzyme activities, decreasing ATP synthesis, and inhibiting protein synthesis.^41^ These consequences result in the impairment of the bacteria's normal metabolic functions, further diminishing their survival capabilities. Due to a compromised skin barrier, wounds exposed to the air become more vulnerable to bacterial infiltration, resulting in infections and delayed wound healing. As illustrated in Fig. 4a and b, the GMH/PDA hydrogel demonstrated remarkable photothermal conversion when exposed to 808 nm NIR irradiation at varying power densities. This phenomenon arises from the absorption of photon energy by the aromatic ring and nitrogen–oxygen functional groups of PDA upon exposure to 808 nm NIR irradiation, leading to an elevation in the internal temperature of the hydrogel. Within a 3 min interval, the hydrogel experienced a maximum temperature increase of 33.9 ± 1.2 °C. Photothermal stability plays a pivotal role in the performance of photothermal materials. As illustrated in Fig. 4c, the temperature of the GMH/PDA hydrogel exhibited a slight increase after 5 “ON/OFF” cycles, potentially attributed to a reduction in the hydrogel's water content resulting from elevated temperatures. To delve deeper into the photothermal conversion capabilities of the GMH/PDA hydrogel, we conducted a heating/cooling experiment involving the hydrogel and water in a controlled environment (Fig. 4d and f). We quantified the photothermal conversion ratio of the GMH/PDA hydrogel as 40.2%, demonstrating similarity to the photothermal conversion ratio of PDA nanoparticles. This suggests that the incorporation of PDA into the hydrogel does not affect its photothermal conversion capability.

### 3.6. The antioxidant capacity of GMH/PDA hydrogel

Wound healing is a complex biological process that relies on the coordinated action of numerous molecules and cells. Oxidative stress commonly occurs during this process, potentially resulting in cellular and tissue damage.^42^ Hence, employing substances with antioxidant capacity is crucial for promoting wound healing. As illustrated in Fig. 4g–j, the GMH/PDA hydrogel exhibits a rapid and concentration-dependent capacity for scavenging free radicals. At a concentration of 40 mg mL^−1^, the hydrogel displayed a scavenging rate of nearly 90% for DPPH, while the scavenging rate for PTIO approached 80%. This indicates that the GMH/PDA hydrogel possesses exceptional antioxidant capacity and can efficiently mitigate inflammatory responses in the context of wound healing.

### 3.7. *In vitro* antibacterial activities

**Fig. 5:**
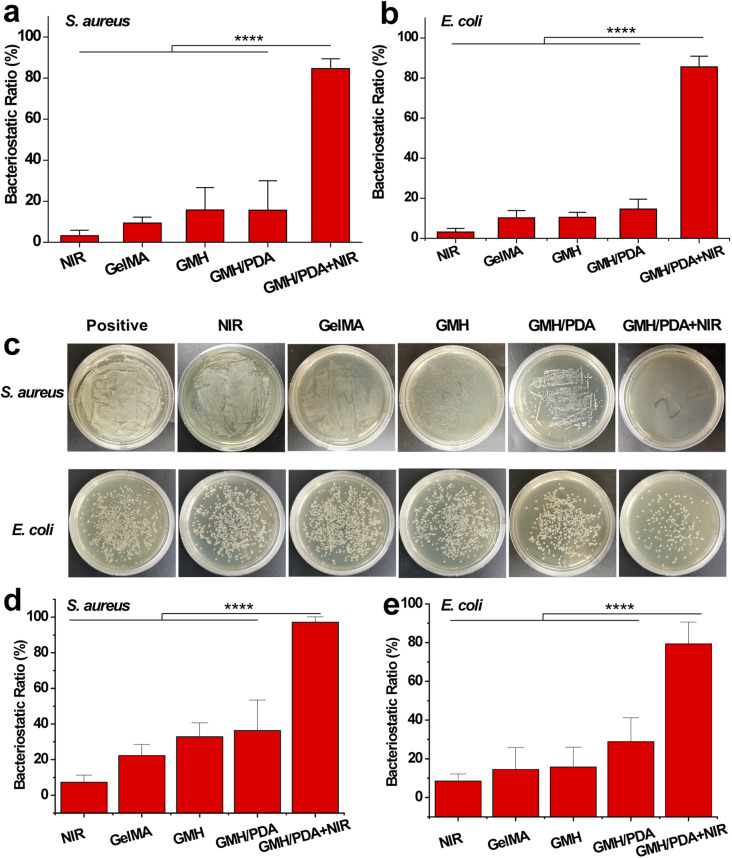
Bacteriostatic effect of different hydrogels towards (a) *S. aureus* and (b) *E. coli*. (c) Representative photographs of bacterial colonies after different treatments towards *S. aureus* and *E. coli*. Bacteriostatic effect of different hydrogels towards (d) *S. aureus* and (e) *E. coli*.

The *in vitro* antimicrobial properties of GelMA, GMH, GMH/PDA hydrogels were compared by liquid antimicrobial assay. Gram-positive (*S. aureus*) and Gram-negative (*E. coli*) bacteria were used for *in vitro* antimicrobial experiments. As shown in Fig. 5a and b, after 12 h of incubation, the GMH group showed 15.8% and 10.5% inhibition against *S. aureus* and *E. coli*, respectively, which was significantly higher than that of the GelMA group (9.4% and 10.2%). The inhibition rates of the GMH/PDA group (15.6% and 14.6%) were similar to those of the GMH group. Interestingly, the GMH/PDA + NIR group showed significantly higher inhibition rates of 84.6% and 85.6% than the other three groups. While no significant antibacterial effect was observed in the NIR group alone. These results indicate that the introduction of OHA improved the antimicrobial properties of the hydrogels, which can be attributed to the aldehyde group in OHA. More importantly, the antimicrobial performance of GMH/PDA itself combined with the photothermal antimicrobial property can effectively improve antimicrobial efficiency. At the end of the incubation, we spread the bacterial suspension on agar plates and incubated it for another 12 h. The growth of the colonies proved that the GMH group had a slight inhibitory effect on *S. aureus* and *E. coli* and was higher than that of the GelMA group, whereas the inhibitory effect of the GMH/PDA group was similar to that of the GMH group (Fig. 5c). Notably, the GMH/PDA + NIR group showed 97.2% and 79.4% inhibition of *S. aureus* and *E. coli*, respectively, which was significantly higher than those of the other three groups (Fig. 5d and e).

### 3.8. Biocompatibility assessment of the hydrogels

**Fig. 6:**
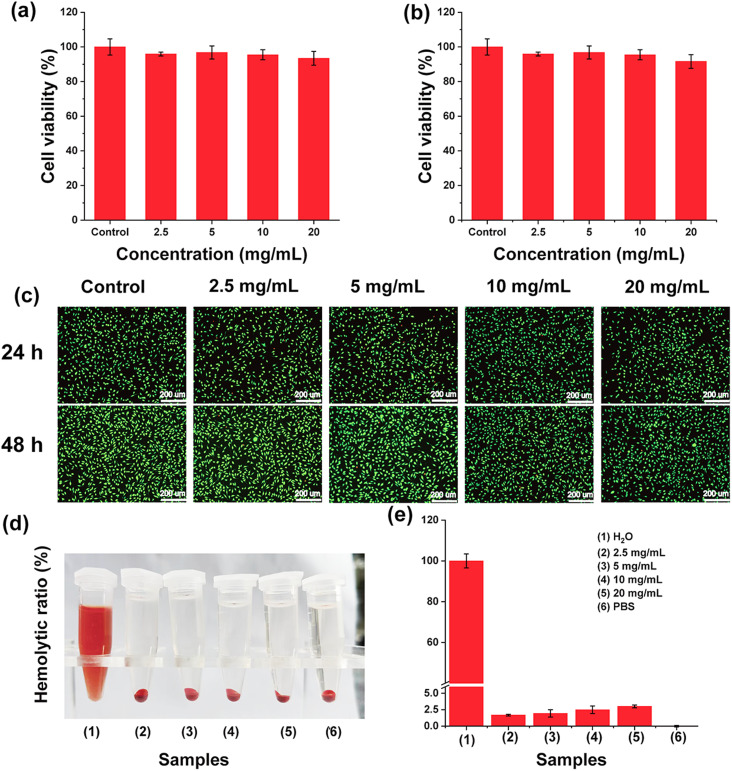
The survival of L929 cells co-incubated with different concentrations of GMH/PDA at (a) 24 h and (b) 48 h. (c) Live–dead staining of L929 cells. (d) Macroscopic photographs of hemolysis experiments. (e) The results of the hydrogel hemolytic activity test.

An important prerequisite for the *in vivo* application of biomaterials is a good safety profile. First, L929 cells were selected as model cells to explore the safety of GMH/PDA hydrogel. As shown in Fig. 6a and b, the viability of all cells co-incubated with the hydrogel extract was greater than 90%. Even at a high concentration of 20 mg mL^−1^, the cell viability of L929 cells at 24 h and 48 h was 93.4% and 91.6%, respectively. The results indicated that GMH/PDA hydrogel would not release any substances with high cytotoxicity and it had excellent cytocompatibility. Similarly, cell live–dead staining presented results similar to those of CCK-8. As shown in Fig. 6c, the absolute majority of the cells were colored green (living cells); very few cells were shown red (dead cells) and none of the living cells changed significantly in morphology. Similarly, the results of CCK-8 and live–dead staining showed that GMH/PDA hydrogel was not significantly toxic to HeCat cells (Fig. S5†). The results of live–dead staining demonstrated in more visual way the high cytocompatibility of GMH/PDA hydrogel. There was negligible thermal damage due to the photothermal effect of the hydrogel.^43,44^

In addition, haemolysis is a common method to characterise blood contact materials. A haemolysis assay was next used to test the blood compatibility of GMH/PDA. The haemocompatibility of the GMH/PDA hydrogel was next tested using a haemolysis experiment, with H_2_O as a positive control and PBS as a negative control. As shown in Fig. 6d, the hydrogel and PBS groups were similar: that is, the supernatant was clear and transparent with erythrocyte precipitation at the bottom. However, the supernatant of the positive control showed a red colour with no precipitate at the bottom, indicating that the erythrocytes had completely ruptured and lysed. Further calculation of the hemolysis rate showed that even with a GMH/PDA concentration of up to 20 mg mL^−1^, the rate of hemolysis was less than 5% (Fig. 6e). The above results indicate that GMH has good cytocompatibility and could be an ideal candidate for wound dressing.

### 3.9. Cell migration

**Fig. 7:**
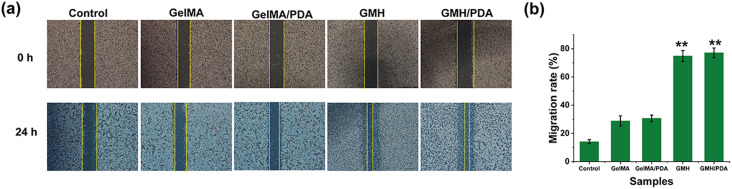
(a) Representative photographs of wound healing experiment for L929 cells and (b) quantification of migration area for L929 cells.

Wound healing is a complex process in which the ability of cells to migrate plays a vital role. Therefore, cell scratch assays were used to assess the ability of GMH/PDA hydrogel to promote cell migration. The migration ability of L929 cells in the different hydrogel groups can be clearly seen in Fig. 7a. The results showed that the migration ability of L929 cells co-incubated with hydrogel extracts was significantly enhanced and the residual area was significantly reduced. Further calculation of the cell migration rate revealed that the cell migration rates of the control, GelMA, GelMA/PDA, GMH and GMH/PDA groups were 14.1 ± 1.4%, 30.7 ± 2.4%, 28.8 ± 3.6%, 74.8 ± 3.9% and 77.1 ± 3.5%, respectively (Fig. 7b). Similar to reports in the literature, GelMA has some ability to promote cell migration. In addition, the migration ability of the cells was significantly enhanced upon further addition of OHA (*p* < 0.01). The results showed that the incorporation of GelMA and OHA in the hydrogel fractions greatly improved the migration of the cells. Notably, research has demonstrated that photothermal therapy forms the foundation of GMH/PDA hydrogel antimicrobial treatment, effectively reducing the risk of wound infection. Nevertheless, its impact on cell migration is relatively limited.^45^ In conclusion, the results of cytocompatibility and cell migration indicate the potential application of GMH/PDA hydrogel in wound dressings.

In addition, we have compared the wound healing performance of the proposed GMH/PDA hydrogel with that of other biomaterials based on PDA NPs (Table 1). It is clear that PDA exhibits superior wound healing ability.

**Table 1:** Comparison of the wound healing capacity of PDA NPs

Material	Wound closure ratio (%)	Ref.
Sodium alginate/PDA hydrogel	74.98 (on day 7)	46
Fibrous electrospun SF/PDA membranes	89.6 (on day 14)	47
Polyacrylamide/PDA/Mg^2+^ hydrogel	79.46 (on day 7)	48
MXene@PDA–cryptotanshinone nanosheets + NIR	∼100 (on day 10)	49
2-Hydroxypropyltrimethyl ammonium chloride chitosan/alginate/PDA/Fe^3+^ nanoparticles	99.6 (on day 15)	50

## 4 Conclusion

In conclusion, GMH/PDA hydrogel was successfully synthesized in this study for eliminating wound inflammation, inhibiting infection and promoting wound healing. Under NIR irradiation, GMH/PDA hydrogel showed excellent antibacterial activity against both Gram-positive and Gram-negative bacteria. In addition, the experimental results of a cell migration assay confirmed that GMH/PDA hydrogel could significantly increase the cell migration rate, thus accelerating the wound healing process. Interestingly, the GMH/PDA hydrogel also showed high antioxidant properties, which helped to reduce the inflammatory response at the wound site. More importantly, this GMH/PDA hydrogel could be degraded and absorbed after wound healing, avoiding additional damage caused by secondary surgery. These results indicate that the GMH/PDA hydrogel has the advantages of eliminating inflammation, inhibiting wound infection, and promoting wound healing, and has good application prospects in clinical applications.

## Conflicts of interest

The authors declare that they have no known competing financial interests or personal relationships that could have appeared to influence the work reported in this paper.

## Supplementary Material

RA-014-D3RA08219A-s001
