# Supplementary material for: Injectable hydrogels doped with PDA nanoparticles for photothermal bacterial inhibition and rapid wound healing in vitro
Source: RSC Adv. 2024 Jan 17;14(4):2778–91. doi: 10.1039/d3ra08219a (PMC10792480; doi:10.1039/d3ra08219a)
Supplement: RA-014-D3RA08219A-s001 [file RA-014-D3RA08219A-s001.pdf]

Electronic Supplementary Material (ESI) for RSC Advances.
This journal is © The Royal Society of Chemistry 2024

Supporting information

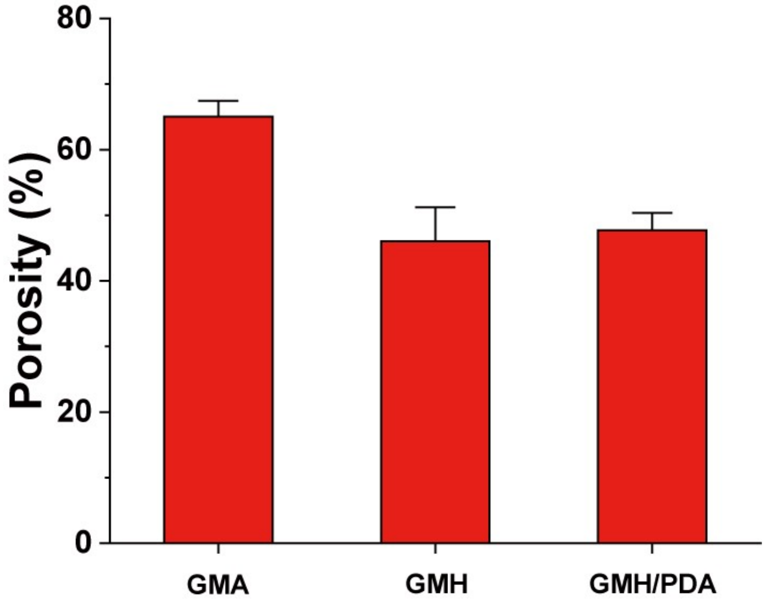

Fig.S1 The porosity of different hydrogels.

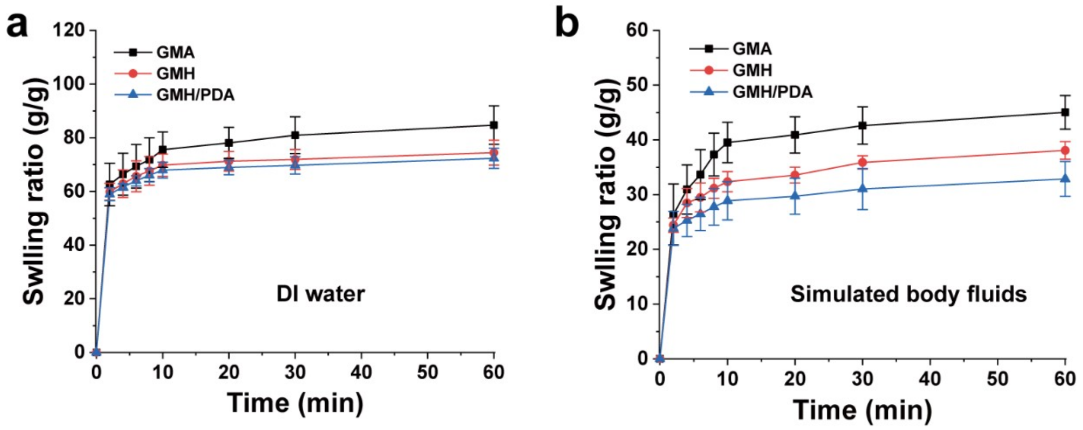

Fig.S2 Swelling kinetics of different hydrogels in DI water; (e) swelling kinetics of different hydrogels in SBF.

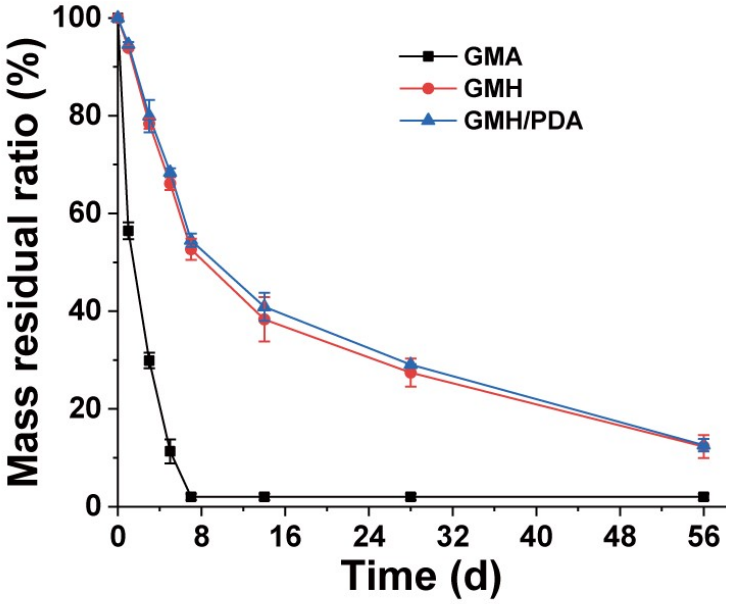

Fig.S3 Degradation curves of different hydrogels in SBF.

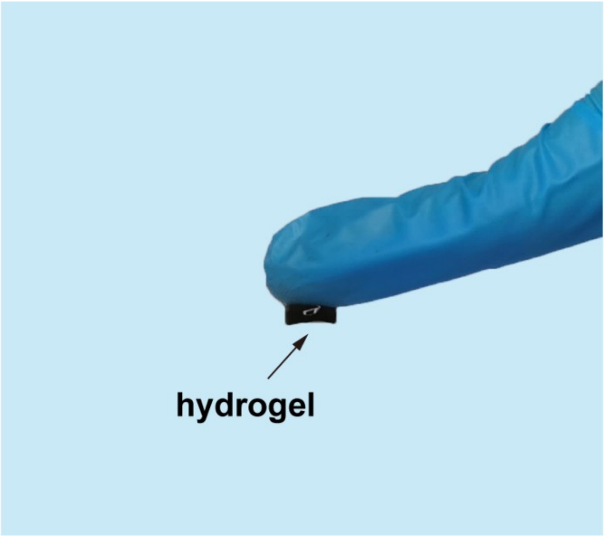

Fig.S4 Hydrogel adhesive digital photo.

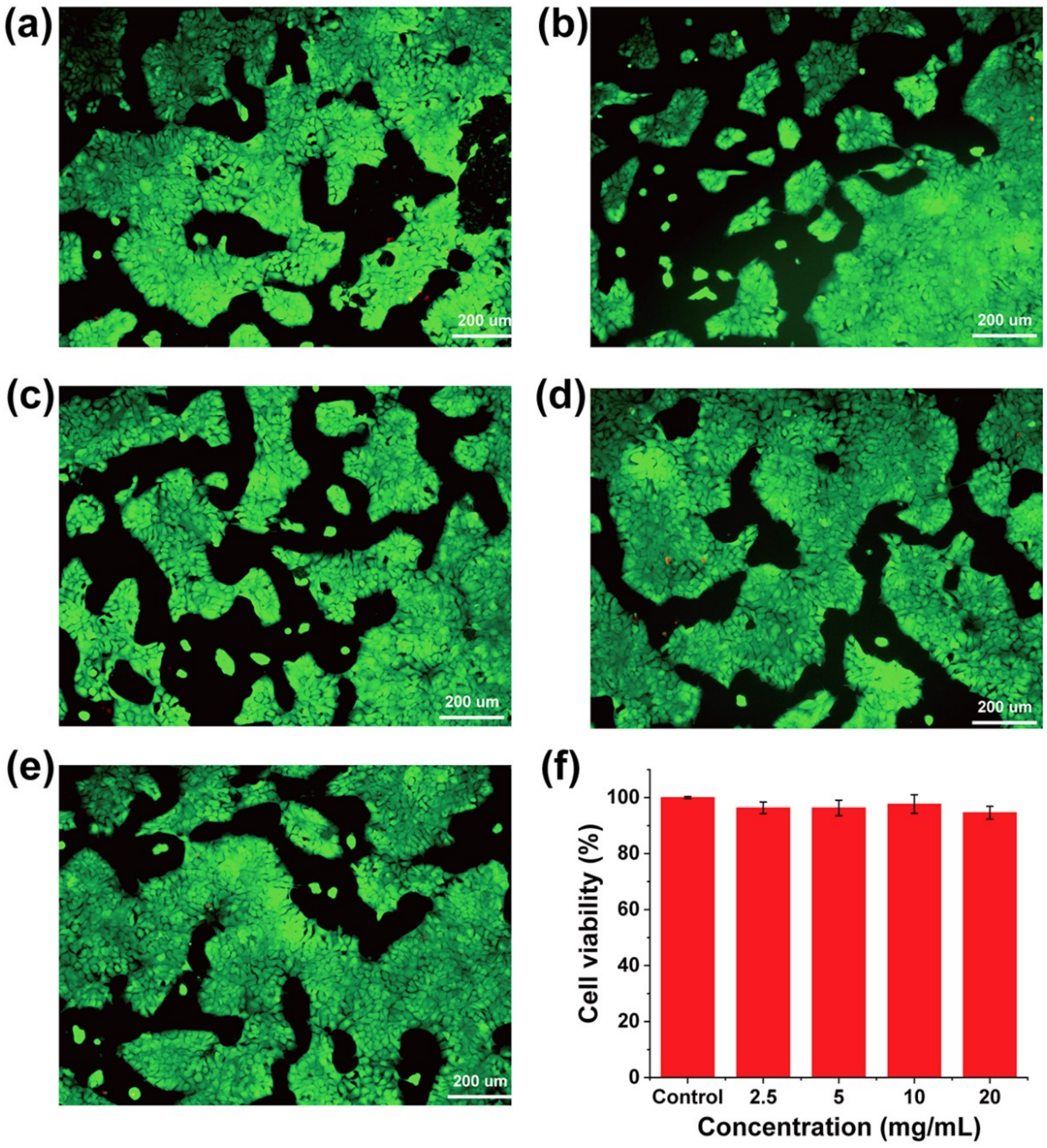

Fig.S5 (a-e) Effects of different concentrations of GMH/PDA hydrogel on the viability of HaCat cells (a Control, b 2.5 mg/mL, c 5 mg/mL, d 10 mg/mL, e 20 mg/mL.); f the survival of HaCat cells co-incubated with different concentrations of GMH/PDA.
